# Impact of Employees' Workplace Environment on Employees' Performance: A Multi-Mediation Model

**DOI:** 10.3389/fpubh.2022.890400

**Published:** 2022-05-13

**Authors:** Gu Zhenjing, Supat Chupradit, Kuo Yen Ku, Abdelmohsen A. Nassani, Mohamed Haffar

**Affiliations:** ^1^Institute for Cultural Industries, Shenzhen University, Shenzhen, China; ^2^Department of Occupational Therapy, Faculty of Associated Medical Sciences, Chiang Mai University, Chiang Mai, Thailand; ^3^Program in Leisure Industry Management, Commercial College, Chinese Culture University, Taipei City, Taiwan; ^4^Department of Management, College of Business Administration, King Saud University, Riyadh, Saudi Arabia; ^5^Department of Management, Birmingham Business School, University of Birmingham, Birmingham, United Kingdom

**Keywords:** employee workplace environment, employees' performance, achievement-striving, striving for achievement, analysis

## Abstract

This study examined the impact of workplace environment on employee task performance under the mediating role of employee commitment and achievement-striving ability. For this purpose, data were collected from the academic staff under a cross-sectional research design, and they were approached through convenience sampling technique. As per recommendations of established sample size criteria, we distributed a sum of 420 questionnaires among the respondents. Among these distributed questionnaires, only 330 were received back. The returned questionnaires were checked for missing and incomplete responses and after discarding the missing responses useable responses were 314 which were used for the data analysis. Data had been analyzed through structural equation modeling (SEM) by using Smart PLS 3. The SEM was done based on measurement models and structural models. The results indicated that a positive work environment had the power to improve employee performance. Similarly, a positive work environment also improved the employee commitment level and achievement-striving ability significantly. Both employee commitment and achievement-striving ability also improved employee performance. While in the case of mediation, it had also been observed that workplace environment triggered employee commitment and employee achievement-striving ability which further improved employee performance.

## Introduction

According to the assumptions of human resource management (HRM), improved performance is accomplished through the employees of the organization. Employees are thus viewed as a valuable asset to every firm to improve performance (1). Before the last decades of the 20th century, the performance was viewed as the result of a mix of aptitude and motivation when given adequate resources, and therefore motivating people became an important aspect of most management. Whenever human resource (HR) is used to its greatest capacity, a business may attain limitless productivity, efficiency, and performance. All employees may not work in the same way since they have distinct working styles. Some personnel have the greatest potential regardless of the reward, whereas others benefit from a boost now and again (2, 3). The employees' performances are determined by their willingness and openness to complete their jobs. Furthermore, if employees are willing and open to accomplish their jobs, it is possible that their productivity will grow, which will contribute to improved performance (4).

Employees, equipment, and supplies, on the other hand, must be provided with the required resources to perform, independent of their talents and expertise (5). “Performance appraisal impacts directly onto highly emotional tasks in professional life, judgment of a person's commitment, and competence,” (6). According to several academics, implementing a well-defined framework for analyzing employee performance is critical to a company's successful operation (7). The major difficulty for businesses, according to (8), has been focused on improving the performance of employees efficiently so that their authenticity remains on top. In other sense, how can businesses use performance evaluation procedures to increase their capacity to discern “excellent” employees (those who perform well) from “poor” employees? Furthermore, according to (9), many crucial variables in the study and implementation of a performance assessment model are still missed, which may explain why there is not currently an integrated approach for assessing employee performance.

The physical and behavioral aspects are the two facets of a healthy working climate. The prior refers to the factors which are linked with the ability of employees to remain physically associated to their workplaces. while the etiquettes of office bearers are influenced by the behavioral aspects of the environment, the workplace environment plays an important role in shaping behaviors of employees individually. Consequently, employees' motivation to work hard, their efficiency and performance are shaped by the influence of the quality of the workplace. Worker' levels of willingness to keep motivated, creative, engaged with colleagues, and loyal to job are all influenced by the factors of workplace environment (10). According to some researchers, this feature of relatedness with workplace environment have mixed beneficial and adverse impacts (11).

The majority of the workplace environments in developing countries are not up to the mark. Unfortunately, most firms consider a safe and healthy work environment to be an unnecessary expenditure and do not invest heavily in sustaining a comfortable working environment (12). For sustainable development, it is vital for any firm to have dedicated employees who are committed to their goals. When people work in groups, there is a possibility that they may behave as if they are entrepreneurs, so every group member engages in as many tasks as possible to demonstrate that he/she is the most promising person in the group. Employee commitment levels boost employee performance in firms which enhance their commitment levels. Previously, firms have given their employees job security to boost their dedication to the firm and efficiency (13). Employee performance is tied to employee commitment. Few academics have argued that each commitment element's psychological status varies from one worker to another (14).

It is supposed that affective commitment as well as employee performance have a positive relationship, suggesting that workers have a belief that their companies would be treating them positively (i.e., fair rehabilitation, involvement in choice determination) could boost interpersonal loyalty of them to the organization and, consequently, enhance their effectiveness (15). Moreover, the workers with a high sense of commitment to the company's goals feel a strong sense of ownership over their responsibilities, while the employees with a lower level of commitment to the company's targets feel no such obligation. Certain research indicates that normative commitment and performance of employees have a negative relationship (16). Employees who have a higher level of organizational commitment find themselves “stuck” in situations where they have little option to quit the organization even if they do not really want to stay. As a result, individuals take their jobs in a less serious manner, and their production suffers (16).

Eudemonia refers to working for and achieving job-related goals, as well as realizing one's maximum potential, and is based on the philosophy of eudemonia drive (i.e., achievement striving). Achievement striving, according to the notion, indicates employees' motivation to take action toward personal greatness (17). On the one hand, the social contact motivates accomplishment seeking by facilitating currently operating and combining for the purpose of fostering creativity and accomplishing work objectives. Achievement striving, on the other hand, is a performance-oriented aim that has a beneficial impact on staff performance (17). Employees are more likely to strive for an outstanding performance if they have a strong accomplishment drive. Employees who have meaningful social connections at work are more likely to be motivated to complete the assignments on time (i.e., achievement striving) (18).

Employees' performance has been evaluated before in different business sectors, leaving behind the gap for a specific sector's evaluation. Moreover, different firm level environmental factors along with job-related factors have been evaluated with specific mediation of employee-related factors such as motivation, adaptability, flexibility, proactivity, skill level, and commitment for evaluating the employees' performance (19). This kind of evaluation left a gap for assessing the specific mediating role of employees' commitment between their workplace environment and performance. Therefore, we utilized the employees' commitment as a potential mediator between employees' workplace environment and employees' performance. Similarly, the role of achievement-striving ability has been utilized as mediator previously along with occupational commitment between social interaction and job performance (18) leaving a gap for evaluating the impact of achievement-striving ability between workplace environment and employees' performance. Therefore, this study was designed to evaluate the mediating roles of employees' commitment and achievement-striving ability.

The impact of employee workplace environment has been studied previously for the evaluation of performance of the employees at different organizational levels but has not been studied among employees of the academic institutes therefore, it posed some questions to address whether it has any impact on the performance of employees of academic institutes or not. The question stated that what role could employee commitment and achievement striving ability of employees could play in the context of academic institute job performance of employees? To answer these questions, this study focused on evaluating the impact of the workplace environment of employees on their performance. The multi-mediation analysis was also carried out in this study to evaluate the aiding role of employees' commitment and achievement-striving ability of employees between workplace environment of employees and their performance.

## Theoretical and Hypothesis Support

Employee performance is achieved through the organization's employees, according to HR management theory (20). To increase the performance, employees are thus considered as a vital asset in any company. Previous to the later decades of the 20th century, performance was considered as a combination of ability and motivation when given sufficient resources, and therefore motivating people, became a key element of the most of the management practices (21). When HR is employed to its full potential, a company may achieve unattainable levels of production, efficiency, as well as performance (22). So, this study gets motivation from HR management theory for evaluating the performance of employees.

The willingness as well as openness of employees to fulfill their work determines their performance. Furthermore, if employees are enthusiastic and motivated to accomplish their jobs, their performance is likely to improve, contributing to increased productivity (23). All this could be achieved under the premises of HRM theory. This study also gets a support from the theory of ecological systems. This theory is also known as “individual theory.” According to this theory, people in a specific environment have a dynamic relationship with their social, physiological, and physical environments. This theory also states that the workplace environments are inter-related in which the job settings are connected with each other and have an effect on activities at workplace in terms of context, time and processes (24). This theory underpins the importance of environment at workplace for the workers and individuals involved in organizational processes.

Once employees get a favorable working environment, then they become more dedicated to their assigned tasks which ultimately improves their performance. So, the ecological systems theory has a lot to offer to shape up the workplace environment. This study also gets support from social exchange theory in which favorable workplace environment provides a sort of motivation to the employees to work better. Such motivational activities in organizations take place having background support of some exchanges socially. The process of social exchange takes place between an organization and its workers indicating that the organization recognizes the contributions of its employees and ensures that they are well-cared for (25). This theory provided the basis for understanding the effect of employee performance in the context of the workplace environment.

Employees, in return, do their best to achieve the targets set by their organizations and they perform better in a given favorable working environment. Thus, a social exchange is in practice for this study. Social exchange theory also provides a basis for employees' commitment as if the workplace environment is favorable and suitable, it develops a sense of trust for the organization among the employees. The employees in exchange show more commitment toward the set targets of the organization. This trust is built as a consequence of management support, and as a result, employees are motivated, which aids in the development of a good attitude toward work, and employee commitment is increased, resulting in improved performance (26). A combination of these theories for evaluating the employees' performance has also been studied before and provided a basis for the conduct of this study.

### Relationship of Employee Workplace Environment With Employees' Performance

Employees spend a major considerable amount of time at work, and their working environment has an impact on their performance in integrated ways (27). Employees who are satisfied with their work environment are more likely to have positive work output. A previous study has revealed that factors which shape up the workplace environment show their impact on the performance of employees (28). They also proposed that future studies on this kind of relationships referring to workplace environment and evaluation of performance could be conducted. A few scholars also encouraged future researchers to conduct comparison studies on private and public organizational levels for impact of workplace environments be on employee' performance (29). The researchers observed that the workplace environment is crucial since staff can work more efficiently doing their jobs in a nice workplace, which leads to higher employees' performance and organization output.

The terms “appealing climate” or “supportive atmosphere” refers to a situation which draws people and motivates them to work by giving them possibilities to accomplish (30). Workers are more willing to integrate their extraordinary use of skills, abilities, and knowledge to achieve success in a welcoming and supportive workplace environment. Employees will be motivated for a number of reasons to accomplish optimal performance and productivity inside a firm; such motivations could be endogenous or exogenous (31). Endogenous motivations help in accomplishing certain difficult tasks and exogenous motivations are the reward which are given in terms of the acknowledgments and the advanced salaries (31).

Another appropriate workplace strategy is to motivate employees to set their goals. Employees' performance improves as a result of this type of incentive program, and the productivity of the company rises (32). Goal setting serves two main functions as follows: First, to improve the behaviors of the individuals; second, to motivate them to work so that they can work effectively and efficiently (33). Generalized objectives are less successful than a particular aim. Furthermore, in contrast to an achievable objective, excellent performance is attained through hard goals. Based on the strategies of providing a better workplace environment to the employees, a few empirical investigations have been done in recent past in different contexts. These studies hinted to explore this kind of relationship even further for establishing this association as a set parameter in achieving improved employees' performance. Therefore, we suggested the hypothesis as given in the following:

**H1:** Employee workplace environment has positive and significant effect on employees' performance.

### Impact of Employee Workplace Environment on Employees' Commitment

According to prior study, the employees' working atmosphere in the firm is vital and also has a significant impact on employees in a variety of aspects (34). If the working environment fails to attract employees and they have a bad perception of many workplace aspects such as sick leave, performance, mental illness, and performance, their demand will ultimately be lowered to a low level, impacting the institution's growth and productivity (35). Employee commitment to the workplace, innovation, efficiency, commitment, and financial wellbeing all benefit from a nice, secure, and reliable workplace, all of which affect the institution's development (36).

When employees work in groups, the individuals behave as if they are entrepreneurs, and every person in the group engages in as many events as possible to demonstrate how he or she is the brightest in the group. Worker level of commitment boost employee productivity in firms which improve their levels of commitment. Companies have traditionally offered job protection to the workers to boost their loyalty toward the company and performance. Employee performance is linked to employee commitment, which has three facets (affective commitment, continued commitment, and normative commitment). It was previously established that the office atmosphere had a favorable influence on workers' commitment to perform (37). As a result of this literature support, we hypothesized the following:

**H2:** Employee workplace environment significantly affects employees' commitment.

### Impact of Employee Workplace Environment on Employees' Achievement-Striving Ability

One of the most critical factors influencing employee performance in an organization is the working environment. In today's competitive corporate world, monetary benefits are insufficient to motivate employees to reach better levels of performance levels (38). A mix of monetary and non-monetary rewards, on the other hand, is more effective better levels of employee performance, which results in increasing of achievement aims of the company for an instance, and it was observed that the employees of certain sector of companies wanted a pleasant, relaxing environment, and to achieve a higher degree of performance, a cooperative working atmosphere is required.

Billings noted that the employees are the focus of organizational decisions as they are present at their workplaces most of the day (39). In contemporary organizations, justice is not always administered through the equal distribution of employment resources as well as the provision of clear and acceptable explanations for choices taken, and employees are not always treated with dignity and respect throughout policy and procedure execution (40). This leads to a worse workplace environment while, it is proven that a better workplace environment is always suitable in achieving something good for the organizations. Achievement striving is totally the drive for achieving the targeted goals by the employees. The employees who are more targeted toward the goals are more productive in terms of their performance (18). In this way, if employees are given suitable workplace environment, then it could initiate achievement-striving ability in employees. So, based on this possible logic, we devised the following hypothesis:

**H3:** Employee workplace environment significantly affects employees' achievement-striving ability.

### Mediation Between Workplace Environment and Employees' Performance

The performance of employees is a popular issue, and this is influenced in a range of ways by the workplace. Behavioral and physical features of a typical working environment are critical. All components which are linked to an employee's ability to physically engage with the workplace are referred to as the physical setting (41). While behavioral environmental components relate workplace occupants' etiquettes with one another. The workplace atmosphere has a positive impact on individual employee behavior (42). Consequently, workplace environment quality has a significant impact on workers and their motivation, enthusiasm, creativity, and efficiency. Work motivation, innovative behavior, attendance, colleagues' engagement, and career management are all influenced by how strongly they are connected to a company (43).

Depending on the physical circumstances in the workplace, it might have a beneficial or harmful impact. The majority of the working environment in underdeveloped nations is insecure and dangerous. However, most businesses consider a safe and healthy work atmosphere to be an absolute waste of money and therefore do not invest extensively in keeping it in good shape (44). Employees working in an unstable and unhealthy atmosphere, putting them at risk for occupational sickness related to the adverse effects of the environment on their productivity, which has an impact on the organization's total productivity (45). Employees are dealing with serious environmental issues at work, particularly in the software business, which makes it difficult to provide necessary amenities to improve their performance level (46).

**Figure 1:**
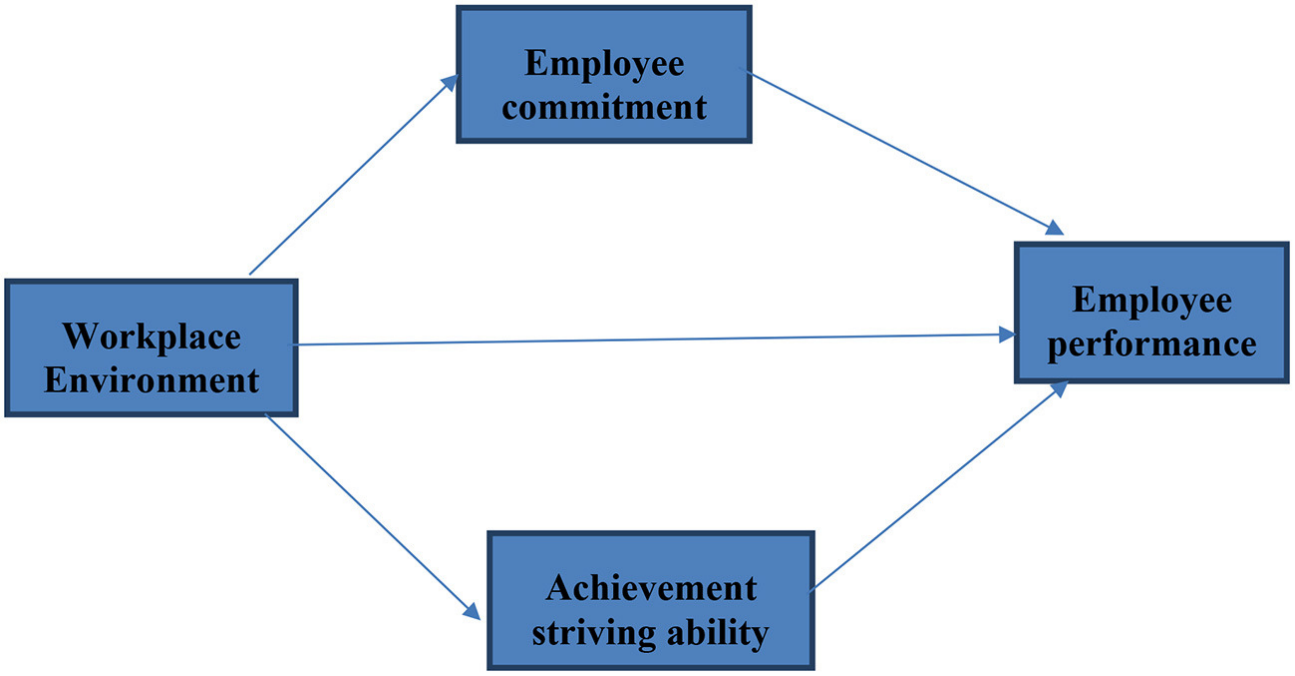
Conceptual framework.

Scholars recently examined software house workers' performance in the presence of physical and behavioral workplace ambient variables (47). As a result, this study's major goal is to analyze and evaluate the factors of the working as well as behavioral environment that influence employee performance. To accomplish the given task of evaluating the impact of the workplace environment of employees' performance, there was a dire need to find the facilitators who could boost the relationship of workplace environment and employees' performance. Based on this need, employee's commitment and achievement-striving ability of employees, which are discussed in previous section, are used as mediators of this study. So, we proposed the following hypothesis (see Figure 1).

**H4:** Employee commitment significantly mediates the relationship between employee workplace environment and employee performance.

**H5:** Achievement striving significantly mediates the relationship between employee workplace environment and employee performance.

## Research Methods

This study used a cross-sectional research design to collect data from the participants. This research design was commonly used in survey research and previously the researchers have used cross-sectional research design in their studies (48, 49). This study design was most suitable to our aim of the study which was to investigate the impact of the workplace environment on employee performance. So, we had obtained the perception of the respondents through a cross-sectional research design. In this regard, teachers from the academic institutes were approached. The respondents in this study were selected based on previous studies, where academic teaching staff were approached for data collection to study the impact of workplace environment (50). Before approaching the academic teaching staff for data collection, we sought formal approval from the administration.

After getting approval from the administration, we contacted the teaching staff according to the list provided by the academic institutes. Moreover, before asking the respondents to provide their feedback, we ensured them regarding data confidentiality and their written informed consent was obtained. Additionally, to increase their motivation in the study, we offered chocolates to the respondent with the questionnaire, so that they could fill out the survey questionnaire with motivation. Before distributing questionnaires to the respondents, a suitable sample size was determined and the criteria regarding setting a reasonable sample size were consulted. In this regard, the widely used and accepted criteria for sample size devised by the study in (51), and previously used by various researchers, were followed (52).

Thus, as per the recommendations of this sample size criteria, we distributed a sum of 420 questionnaires among the respondents and of these distributed questionnaires, only 330 were received back. The returned questionnaires were checked for missing and incomplete responses and after discarding the missing responses 314 were retained. Additionally, we have employed Smart PLS software, which handles the small sample size very comfortably, so the issue of sample size does not raise any question in this study (53).

Owing to the cross-sectional nature of the study, it was likely to encounter common method bias in this study. We employed several techniques to reduce the issue of common method bias, we interchanged the place of the scales and items in the questionnaires, so that respondents could not develop a correlation among the study constructs while reporting the responses. This helped us to reduce common method biases (54, 55).

### Demographic Characteristics

The first section of the questionnaires dealt with demographic characteristics related to qualification, gender, and teaching experience. From the perspective of qualification, respondents were mostly with 18 years of education and 16 years of education; however, the percentage of 18 years education among respondents was high (90%). Second, the distribution of the respondents according to gender's perspective was almost equal [i.e., 54% (male) and 46% (female)]. While most of the teaching staff were employed in service with experience of more than 3 years, very few have <1 year of experience.

### Instrument Development

We followed a five-point Likert scale to collect the data for all exogenous and endogenous constructs ranging from five to one on a description of strongly agree to strongly disagree. The independent variable in this study (workplace environment) is measured through 10 items. The one-dimension of the environment (hedonic environment) is used in this study, which denotes the positive side of the workplace environment. Sample items for this scale include, “The transparency of rules in my institution is making my work easier,” and “My company is a positive workplace.” This scale is used in a recent study (50). This scale contains reverse coded questions, and we have also used these reverse coded questions to restrict the respondents from providing monotonic responses. The outcome variable in this study, employee performance is measured through six-items scale covering the perception of employees' task performance. This scale is developed by Koopmans et al. (56). The sample items for this scale include, “I kept in mind the results that I have to achieve in my work.” Although in previous studies (50), another dimension of employee performance has also been used such as contextual and counterproductive work behavior. However, we have used task performance as a measure to assess the response regarding employee performance which denotes it well.

Employee commitment is assessed based on six items-based scale of affective commitment developed by a research team (57) with sample item, “I would be happy to work at my organization until I retire.” While the second mediating variable, achievement-striving ability is assessed based on a scale developed by in a study (58) with five-items scale. A sample item for this scale, includes, “I am a very determined person when it comes to my job.”

## Results

### Assessment of Measurement and Structural Model

We have employed a multi-variate data analysis tool in this study to test the hypotheses through structural equation modeling (SEM). For this purpose, the most commonly used partial least square (PLS) approach through Smart PLS was used (59). This software deals very well with the complex nature of research frameworks/models (60). In addition to this, smart PLS has good predicting capability even with a small sample size and it deals with small sample size very well. Lastly, it does deal better with the non-normal data and the issue of normality is handled by Smart PLS very well. Assessment of SEM is based on two approaches/methods, the first one is based on the measurement model while the second one is based on structural model (61).

Table 1 illustrates the reliability and validity of the study constructs, based on the assessment of the measurement model. At this stage of reliability and validity of the study, the model has been confirmed. For the issue of reliability, the first measure in this regard that is used is Cronbach Alpha or is termed as alpha. The minimum acceptable value for this indicator of reliability is 0.60 (60, 62). Alpha statistics have been found statistically high above this benchmark; for instance, the alpha value for the construct, workplace environment is 0.929, for employee performance it is 0.745, for achievement-striving ability it is 0.839 and for employee commitment, it is 0.893. Thus, all the constructs possess good alpha reliability. Similarly, the second measure of reliability (rho-A) is also within the acceptable range (>0.60). The value of Rho-A for the workplace environment is 0.939, for the employee performance is 0.768, for the achievement-striving ability is 0.877, and for the employee commitment is 0.925. Thus, the second measure of reliability is also met. The third measure of reliability is based on composite reliability, which also shows a good level. The values for composite reliability are within a range of 0.830–0.941, illustrating good composite reliability.

**Table 1:** Reliability and validity of the study constructs.

**Construct**	**Cronbach's**	**rho_A**	**Composite**	**AVE**
	**alpha**		**reliability**	
Achievement-striving ability	0.839	0.877	0.887	0.663
Employee commitment	0.893	0.925	0.918	0.653
Employee performance	0.745	0.768	0.830	0.551
Workplace environment	0.929	0.939	0.941	0.641

In the case of validity, it has been tested through average variance extracted (AVE) and it has been found that the AVE of the respective constructs is greater than the threshold limits of the acceptable range (≥0.50). All the study constructs possess greater AVE values (≥0.50) which indicate that the convergent validity has been established (63) as illustrated through Table 1. The AVE values range between 0.551 and 0.663.

**Figure 2:**
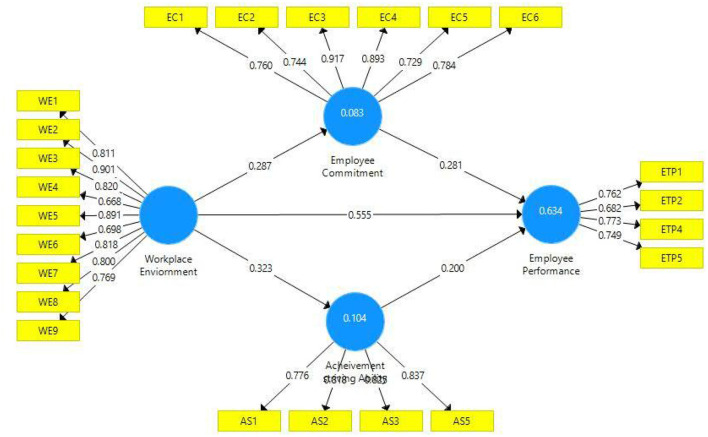
Path estimates.

The second measure to assess the convergent validity is outer loadings (Figure 2). At this stage, each indicator was checked for outer loadings, and it was observed that outer loadings are above the threshold value of 0.708. Table 2 illustrates the outer loadings of all study constructs. Two items have been dropped in this study due to weak or poor outer loadings. One item from the study constructs workplace environment (WE-10). Similarly, from employee performance, two items (ETP-3 and ETP-6) have been dropped due to poor outer loadings. One item from the construct achievement-striving ability (AS-4) was dropped. One item with slightly low outer loading (ETP-2) was retained in employee performance as the AVE of this construct was above the threshold value (≥0.50). Thus, all the indicators met with convergent validity criteria, and it can be referred that the model possesses convergent validity.

**Table 2:** Outer loadings (convergent validity).

**Items**	**Achievement-**	**Employee**	**Employee**	**Workplace**
	**striving ability**	**commitment**	**performance**	**environment**
AS1	0.776			
AS2	0.818			
AS3	0.825			
AS5	0.837			
EC1		0.760		
EC2		0.744		
EC3		0.917		
EC4		0.893		
EC5		0.729		
EC6		0.784		
ETP1			0.762	
ETP2			0.682	
ETP4			0.773	
ETP5			0.749	
WE1				0.811
WE2				0.901
WE3				0.820
WE4				0.668
WE5				0.891
WE6				0.698
WE7				0.818
WE8				0.800
WE9				0.769

While testing the other side of validity (discriminant validity), we have followed two well-established criteria, the first one is Fornell and Larcker (64) and Heterotrait-monotrait (HTMT) ratio of correlations ratios (60). Tables 3, 4 illustrate these two criteria. The first criteria in this regard indicates that the square root of the AVE of variables is higher than the correlations among them (52, 65). For instance, the square root of AVE of achievement-striving ability is 0.814 which is higher than the correlations in that column (bold and underlined values in diagonal). Similarly, the square root of AVE of employee commitment is 0.808 which is also higher in that column. Same pattern is observed for employee performance and workplace environment.

**Table 3:** Discriminant validity (Fornell–Larker-1981 criteria).

**Construct**	**Achievement-**	**Employee**	**Employee**	**Workplace**
	**striving**	**commitment**	**performance**	**environment**
	**ability**			
Achievement-striving ability	**0.814**			
Employee commitment	0.401	**0.808**		
Employee performance	0.492	0.521	**0.742**	
Workplace environment	0.323	0.287	0.701	**0.801**

*Note: Values in the diagonal and bold are square root of AVEs*.

**Table 4:** Discriminant validity (HTMT).

**Construct**	**Achievement-**	**Employee**	**Employee**	**Workplace**
	**striving**	**commitment**	**performance**	**environment**
	**ability**			
Achievement-striving ability	-	-	-	-
Employee commitment	0.450	-	-	-
Employee performance	0.573	0.635	-	-
Workplace environment	0.347	0.300	0.723	-

The HTMT ratio is used as the second measure to assess the discriminant validity. Two criteria were observed in this regard (liberal and conservative). Both criteria were met as the values of HTMT ratios in all columns are <0.90 and 0.85, describing that both liberal and conservative criteria are met. Liberal criteria HTMT ratio indicates that value of HTMT should not be higher than 0.90 while conservative criteria indicate that value of HTMT should not be higher than 0.85. Table 4 illustrates the discriminant validity through HTMT ratios.

Two criteria were used to assess the model fitness, namely, the coefficient of determination (*R*^2^) and effect size (*f*^2^). Table 5 illustrates the quality criteria based on coefficient of determination. Here, it has been observed that predictors (workplace environment) along with the mediators (achievement-striving ability and employee commitment) are explaining 63% variation in employee performance; thus, predicting a good and reasonable model fitness (52, 66). Similarly, 10% change is observed in achievement-striving ability and 8% change in employee commitment as a result of the workplace environment. Figure 2 also illustrates the coefficient of determination, and it can be assumed that these values of coefficient of determination are satisfactory (60). Table 6 illustrates the effect size in terms of *f*^2^. All the effect sizes have been found satisfactory and depict good quality criteria (52). In addition to this, we have also tested the model predictive relevance based on *Q*^2^ (67) and all the values of *Q*^2^ have been found to be higher than zero, indicating model predictive relevance.

**Table 5:** Coefficient of determination.

**Endogenous construct**	***R*** **^2^**	***R*** **^2^ adjusted**
Achievement-striving ability	0.104	0.101
Employee commitment	0.083	0.080
Employee performance	0.634	0.630

**Table 6:** Effect size.

**Construct**	**Achievement-**	**Employee**	**Employee**
	**striving**	**commitment**	**performance**
	**ability**		
Achievement-striving ability	-	-	0.086
Employee commitment	-	-	0.175
Workplace environment	0.116	0.090	0.729

### Hypotheses Testing

**Figure 3:**
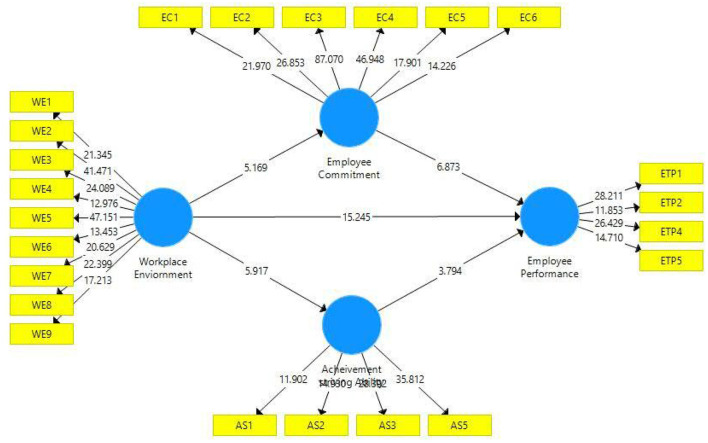
Path significance.

At the final stage, we tested hypotheses based on *t*- and *p*-statistics (See Figures 1 and 3). Direct hypotheses have been tested based on direct paths while hypotheses related to the mediation relations have been tested based on indirect paths (indirect effects). Table 7 illustrates direct, indirect, and total paths while Table 8 indicates hypotheses testing status. The first hypothesis of this study (H1) related to the relationship of the workplace environment and employee performance has been found statistically significant based on *t*- and *p*-statistics and it is accepted. The regression coefficient in this regard indicates that one unit change in the workplace environment will bring 0.55 unit change in employee performance. Moreover, this path also indicates that in the presence of positive workplace environment, employee performance (task performance) moves upward and positive change in task performance is observed.

**Table 7:** Direct, indirect, and total path estimates.

	**β**	**SD**	***t***	***p***
**Direct path**				
Achievement-striving ability -> Employee performance	0.202	0.053	3.794	0.000
Employee commitment -> Employee performance	0.282	0.041	6.873	0.000
Workplace environment -> Achievement-striving ability	0.323	0.055	5.917	0.000
Workplace environment -> Employee commitment	0.289	0.056	5.169	0.000
Workplace environment -> Employee performance	0.555	0.036	15.245	0.000
**Indirect path**				
Workplace environment -> Achievement-striving ability -> Employee performance	0.065	0.020	3.172	0.002
Workplace environment -> Employee commitment -> Employee performance	0.082	0.020	3.998	0.000
**Total path**				
Workplace environment -> Employee performance	0.701	0.029	24.444	0.000

**Table 8:** Hypotheses testing.

		**Coefficient (β)**	**SD**	***t***	***p***	**Status**
**Hypotheses**						
H1	Workplace environment -> Employee performance	0.555	0.036	15.245	0.000	Supported
H2	Workplace environment -> Employee commitment	0.289	0.056	5.169	0.000	Supported
H3	Workplace environment -> Achievement-striving ability	0.323	0.055	5.917	0.000	Supported
**Mediation hypotheses**						
H4	Workplace environment -> Employee commitment -> Employee performance	0.082	0.020	3.998	0.000	Supported
H5	Workplace environment -> Achievement-striving ability -> Employee performance	0.065	0.020	3.172	0.002	Supported

Similarly, the second hypothesis (H2) of this study which is based on the relationship of the workplace environment and employee commitment has also been found statistically significant as evident from the *p*- and *t*-statistics (H2 supported). This state of affairs indicates that with the upward change in the workplace environment there will be positive change in employee commitment. It can be safely assumed that a positive workplace environment tends to promote employee commitment. The third hypothesis of this study was based on the relationship of the workplace environment and achievement-striving ability, which has also been found statistically significant as illustrated in Table 8 (H3 is supported). Thus, a positive change in the workplace environment increases the achievement-striving ability of the employees at the workplace.

While talking to mediation hypotheses, these have been tested through the indirect effects as illustrated in Table 7. Indirect effect for the path Workplace Environment → Employee Commitment → Employee Performance has been found statistically significant (*p* < 0.005) which indicates that employee workplace environment positively increases the employee commitment level which further triggers employees to demonstrate a higher level of employee performance (H4 supported). Similarly, the indirect effect in H5, i.e., Workplace Environment → Achievement striving Ability → Employee Performance has also been found statistically significant (*p* < 0.05) (H5 supported). This indicates that a positive workplace environment improves employees' achievement-striving ability which further enhances their ability to show a higher level of performance.

## Discussion

This study analyzed the impact of employee workplace environment on employee related factors including employee commitment and achievement-striving ability of the employees. The hypothesis of this research indicated that the workplace environment had a significant impact on shaping the performance of employees. A lot of research in the past had evaluated the similar kind of relationships in which changing environments and the factors of environments of workplace had significant contribution toward the job performance of employees (68). For instance, the work in (10) stated that with an increase in per unit variance for physical and behavioral environmental factors, employee's performance was increased which supported our argument. The possible reason behind this outcome was the psychological ability of employees which molded or reshaped the behaviors of employees in case of conducive and restrictive environments of workplace. All employees may not work in the same way since they have distinct working styles due to different workplace environments. Some personnel have the greatest potential regardless of the workplace conditions, whereas others benefit from a supportive environment of the workplace (2).

The direct effects of workplace environment of employees on employee commitment and achievement-striving ability were also evaluated in this study and found significant outcomes indicating that workplace environment influences the employee-based factors as well. The direct effects on employee commitment showed that if a conducive environment at the workplace was provided, then it could develop a stronger sense of commitment in the employees toward their job and organizations. Similar kind of results were also reported by some of the previous researchers (69). In exploration of the relationship between workplace environment with employee commitment, these researchers found that if environment of workplace is suitable then it could lead to wellbeing of employees which in turn improve commitment to work by the employees. Employee commitment levels boost employee performance in firms that increase their commitment levels. Previously, companies have given their employees job security to boost their dedication to the firm and performance (13).

Another dimension to this study was exploration of the relationship between workplace environment and achievement-striving ability of employees. The results indicated similarly the positive association between workplace environment and achievement-striving ability of employees. This kind of relationship evaluation was new as no one in past had evaluated the direct association of workplace environment of employees to achievement-striving ability of employees. The majority of the workplace environment in underdeveloped countries is not safe. Unfortunately, most firms consider a safe and healthy work environment to be an unnecessary expenditure and do not invest heavily in providing a comfortable working environment (12). The indirect effects of employee commitment and achievement-striving ability between workplace environment of employees and their performance are also evaluated in this study.

Both indirect effects of employee commitment and achievement-striving ability proved to be significantly mediating the relationship of workplace environment of employees and employee performance. This indicated that if employees were more committed to their work, then association of workplace environment and employee performance would be enhanced. Similarly, if employees had good ability of achievement striving then association of workplace environment with employees' performance would also be strengthened. Few researchers have claimed that the psychological status of every commitment element differs from one employee to the other (14). It is assumed that organizational commitment and employee performance have a positive relationship, implying that employees who perceive a firm's behavior toward companions is decent (i.e., humane treatment, involvement in judgment) might very well boost their sentimental commitment with the organization and their performance in the organization (15). The results of the this study are related to the work discussed in (18) but with a limitation that they evaluated the mediating link of employee commitment along with some moderators as well.

### Theoretical and Practical Implications

From a theoretical perspective, this study tends to add to the existing body of knowledge by investigating the impact of a positive work environment on employee performance which is the contribution of the study. Moreover, this study has tested two mediating mechanisms and proved that achievement-striving ability and employee commitment as a mediator increase employee task performance, which is also a unique contribution. The perception of academic staff has been documented in this study which is the contribution of the study. From the practical point of view, this study advocates that organizations should focus on the creation and provision of a positive workplace environment at the workplace to improve the task performance of the employees. Similarly, a positive work environment promotes the achievement-striving ability of the employees, so organizations should also focus on improving the achievement-striving ability of the employees through a positive workplace environment.

### Limitation of the Study

Just like other studies, this study has also some limitations. The first limitation is its cross-sectional nature, which does not allow us to assume cause and effect relationships. In the future, researchers should focus on other research designs in replicating this model, which might provide deeper insights into longitudinal research design. Second, only academic staff were approached for data collection; in the future, considering other sectors can provide useful insights. Particularly, banking sector employees can be approached in future studies. Third, we have anticipated only one side of a workplace environment, while in the future, other types of workplace environments should also be tested. Further, this study in future opting larger sample size can provide more detailed and deeper insights regarding the relationship between the workplace environment and employee performance. We have used two mediating mechanisms in this study, considering other mediating variables such as job satisfaction can also be a future avenue. This model can also be tested with the moderating phenomenon in the future such as leadership styles or cultural variables such as power distance and collectivism.

## Conclusions

Based on the empirical findings of this study, it can be concluded that a positive work environment promotes employee performance within organizational circuits. More specifically, the workplace environment can improve the achievement-striving ability of the employees, and employees tend to bounce back in difficult situations. Similarly, a positive work environment provides a nurturing and pleasant work environment which promotes employee commitment and employees tend to be loyal to their organizations. In addition to this, it can also be concluded that the employee commitment has the potency to enhance the task performance of the employees; because employees show a higher level of task performance when they are committed to their employer or organization. Similarly, employees with higher achievement-striving ability tend to show a higher level of task performance even in difficult situations. Further it can be endorsed that motivational activities in organizational cultures are triggered under social exchanges, and positive behaviors at workplace are promoted in shape of employee commitment. This increased commitment can result in enhanced and improved individual and organizational performance.

## Data Availability Statement

The original contributions presented in the study are included in the article/supplementary material, further inquiries can be directed to the corresponding author/s.

## Author Contributions

GZ: initial and final draft. SC and KK: analysis and interpretation. AN and MH: proof read, revision, and data validation. All authors contributed to the article and approved the submitted version.

## Funding

Researchers Supporting Project number (RSP-2022/87), King Saud University, Riyadh, Saudi Arabia.

## Conflict of Interest

The authors declare that the research was conducted in the absence of any commercial or financial relationships that could be construed as a potential conflict of interest.

## Publisher's Note

All claims expressed in this article are solely those of the authors and do not necessarily represent those of their affiliated organizations, or those of the publisher, the editors and the reviewers. Any product that may be evaluated in this article, or claim that may be made by its manufacturer, is not guaranteed or endorsed by the publisher.
